# From data to medical context: the power of categorization in healthcare

**DOI:** 10.3389/fmed.2025.1575195

**Published:** 2025-06-11

**Authors:** Batoul Msheik, Hamid Mcheick, Sara Hariri, Mehdi Adda, Mohamed Dbouk

**Affiliations:** ^1^Computer Science Département, Université du Québec à Chicoutimi, Chicoutimi, QC, Canada; ^2^Département de Mathématiques, Informatique et Génie, Université du Québec à Rimouski, Rimouski, QC, Canada; ^3^Computer Science Department, Université Libanaise, Hadath, Lebanon

**Keywords:** context categorization, context of healthcare domain, telemedicine, chronic disease, clinical support

## Abstract

In the rapidly evolving healthcare domain, the ability to structure and interpret contextual medical data is crucial for delivering personalized and efficient patient care. While many existing studies attempt to define medical context through diverse categorizations, they often lack completeness or applicability in the real-world healthcare domain. This paper introduces a novel and comprehensive context categorization model composed of fifteen well-defined categories, bridging the gap between theoretical models and practical requirements in telemonitoring systems for chronic disease management. By incorporating important but often overlooked components such as social determinants, Service Level Agreements (SLAs), and environmental factors our model enhances clarity and strengthens decision-making in clinical settings. We validate the applicability of this framework through detailed case studies on asthma, COPD, and cardiovascular diseases, demonstrating its utility in enhancing telehealth solutions and aiding early intervention strategies.

## 1 Introduction

The development of technologies and the management of knowledge have improved many human life routines and practices in education, finance, industry, and healthcare (1, 2). Healthcare incorporates such technologies as information and communication tools, artificial intelligence and its successors, such as machine learning and deep learning, computing devices, and sensors (3, 4). In other words, combining technology and healthcare improves services for individuals and society (2, 4). Electronic health records (EHRs) (5), smart wearables (6), mobile health applications (7), decision support systems (8), and a variety of emerging technologies that can collect, store, and even analyze health data in real time are part of the integration between technology and healthcare (9). The healthcare domain has grown rapidly over the last ten years and can now support the provision of high-quality care to patients, satisfying both patients and healthcare professionals and reducing healthcare consumption and costs (10). Contextual characteristics play a central role in the effectiveness of healthcare, emphasizing the necessity of thinking about the definitions and dimensions of context in designing a healthcare domain model. Context is the key element of successful computing in the telemedicine domain. In medical domains, context refers to the relevant data that relate to a patient, such as medical history, symptoms, risk factors, and medications. It also allows more precise diagnoses and establishes more powerful treatment plans (11).

The existing categories of context in healthcare organizations are not very efficient for retrieving information that the user’s needs and demands require (12). Moreover, structured medical terminologies such as SNOMED CT provide standardized clinical vocabularies (1), they primarily focus on clinical documentation, and functional status. These systems are not designed to organize contextual information dynamically for use in real-time decision-making, and AI-driven health applications. Our proposed categorization may fills this gap by offering a structure that integrates social, environmental, technological, and temporal dimensions elements that are often missing or scattered across current frameworks. In addition, the context may vary from one field to another, making a standardized approach to understanding and applying context in a specific domain (13). Accordingly, capturing the needs and preferences that provide a complete and clear picture of the patient’s information demand is crucial (14). Making a more meaningful contribution in this research area requires a better understanding of the most critical aspects of context in the healthcare domain. This approach aims to utilize the most relevant data that may improve an individual’s health outcomes and quality of life (15). Its main purpose is to understand and identify the context and provide a generic model for monitoring daily life activity and handling emergency situations for a patient. This paper focuses on context categorization in healthcare, proposing a fifteen-category framework and applying it to three chronic diseases (COPD, asthma, and cardiovascular diseases). In the medical field, context is key for getting diagnoses right, predicting outcomes accurately, and treating correctly.

### 1.1 Research gap and novelty

Existing studies on medical context categorization have laid the groundwork for intelligent health monitoring systems, yet they fall short in several areas. Some existing Models lack specific healthcare parameters or crucial dimensions like social determinants of health, Service Level Agreements (SLA), or real-time medical data. Therefore their applicability in developing robust, responsive, and personalized telemonitoring systems remains limited.

### 1.2 Motivation and contribution

The need for a unified and detailed context categorization framework in healthcare remains unmet. Existing models often lack medical specificity, adaptability, or completeness. This study makes three key contributions:

- Introduce a fifteen-category context model tailored for chronic disease management.
- It extends existing frameworks by incorporating critical dimensions such as Service Level Agreements (SLA) and social context, while also refining other categories through the inclusion of additional contextual entities.
- Applies the model to three prevalent chronic diseases demonstrating their practical relevance and adaptability.

The article consists of five parts. The first presents the existing context categorizations. The second part is an overview of noncommunicable diseases. The third describes our perception of medical concepts. The fourth part provides a case study of designing healthcare systems for chronic diseases (i.e., COPD, asthma, and cardiovascular diseases). The final part discusses this research work and offers the authors’ perspectives.

## 2 Medical context in healthcare

### 2.1 State of the art

Weiser defines context as “all the information that should be taken into consideration for an adjustment.” While this is a helpful starting point, it is too abstract to apply directly in healthcare. Our approach builds on this idea by identifying and organizing the key context elements that matter most for healthcare delivery, monitoring, and personalized care. To address this issue, researchers have proposed various definitions of “context,” some of which focus on listing specific contextual information, such as location, time, and environment. Lacking standard definitions for this term, some useful characteristics can emerge from some of its most common features. While everyone has a general idea of what context is, finding a precise definition is not easy (see Table 1).

**Table 1:** Dimensions context.

Authors	Dimension
Gwizdka (2000) (16)	Internal and external
Gross and Specht (2001) (17)	Location, identity, time, environment, or activity
Antti Aaltonen (2002) (18)	Location, target, calendar, address book, users nearby, history, profile, direction, and speed
Prekop et al. (2003) (19)	Physical and logical
Mayrhofer (2004) (20)	Geographical, physical, organizational, social, emotional, user, task, action, technological, and time
Bunningen et al. (2005) (21)	Operational and conceptual
Miao et al. (2006) (22)	Sensed, profiled, and derived
Chong et al. (2007) (23)	Computing, physical, history, identity, and time
Miraoui and Tadj (2008) (24)	Trigger information and quality-changing information
Arianti Kurti (2009) (25)	User’s profile, activity, and location/environment
Soylu (2009) (26)	User and environment
Zhong (2009) (27)	User, system, environment, social, and time
Tamine et al. (2010) (28)	User, platform, and environment.
Rizou et al. (2010) (29)	Low level and high level
Nageba E. (2011) (30)	Physical and abstract
Kim et al. (2012) (31)	5W1H (Who, When, Where, What, Why, and How)
Bin Guo (2013) (32)	Individual, social, and urban context
Boughareb et al. (2014) (28)	Device, task, user, document, spatiotemporal, environmental, and event
Meshali et al. (2015) (33)	Unusual behavior
Banhato et al. (2015) (34)	Social-demographic factors, such as physical, emotional, and cognitive
Zhang et al. (2016) (35)	Vital signs, medical symptoms, risk factors, activities, and environment
Ameyed (2016) (36)	Time, space, and purpose. Psychological Identity, location, status (physical parameter, medical device), and time
Ahmed et al. (2017) (37)	Individual, medical, auxiliary, location, device, activity, and environmental
Neumuth et al. (2018) (38)	Quality of patient life
Almobarak and Jaziri (2019) (39)	Patient profile, time, location, environment, and social factors
Ajami et al. (2018) (40)	Medical history, disease, treatment, person, technology, task, and event Organization and policies
Taiwo et al. (2020) (41)	Physical factors
Kayes et al. (2020) (42)	Patient information in smart space
Kouamé et al. (2022) (43)	User, resource, and environment

### 2.2 Review state of the art

Many researchers have proposed different ways to categorize medical and general context information. For example, Gwizdka introduced a basic distinction between internal context (related to the user, like emotions or thoughts) and external context (environmental factors) (16). Gross and Specht suggested five key context categories: location, identity, time, environment, and activity (17). Aaltonen extended this by including calendar data, user profiles, nearby users, direction, and speed (18). Prekop et al. proposed two types of context: physical context, which refers to concrete and observable factors, and logical (or abstract) context, which includes internal and mental states (19). Mayrhofer expanded the scope further by including dimensions such as organizational, social, emotional, user, task, action, technological, and time (20). Bunningen et al. organized context into two layers: operational (raw, collected data) and conceptual (interpreted or abstracted information) (21). Miao et al. introduced three types of context: sensed (captured directly), profiled (based on past data), and derived (inferred from combinations) (22). Chong et al. categorized context into computing, physical, history, identity, and time (23). Miraoui and Tadj divided contextual information into trigger information (which initiates automatic services) and quality-changing information (which affects the format or quality of service delivery) (24). Kurti focused on three key areas: the user profile (e.g., age, gender), the user’s activity, and the location or environment (25). Soylu grouped context into two main dimensions: user factors (preferences, physical/mental traits) and environmental factors (location, time, lighting) (26). Zhong proposed five dimensions: user, system, environment, social, and time (27). Tamine et al. classified context into user, platform, and environment (28). Rizou et al. distinguished between low-level context (basic sensor data) and high-level context (interpreted information) (29). Nageba presented a simple classification: physical and abstract context (30). Kim et al. introduced the well-known 5W1H model—Who, What, When, Where, Why, and How—to represent minimal, yet complete, context dimensions (31). Guo considered three levels: individual, social, and urban context (32). Boughareb et al. proposed a more extensive taxonomy including device, task, user, document, spatiotemporal data, environment, and event (28). Meshali introduced unusual behavior as a contextual element (33). Banhato emphasized sociodemographic context, covering physical, emotional, and cognitive factors that affect health (34). Zhang et al. introduced a healthcare-focused model using vital signs, symptoms, risk factors, activities, and environment (35). Ameyed described context in terms of time, space, purpose, and psychological aspects (36). Ahmed et al. identified individual, medical, auxiliary, location, device, activity, and environmental components (37). Neumuth et al. added quality of patient life as an important category (38), while Almobarak presented context as patient profile, time, location, environment, and social factors (39). Ajami et al. proposed a comprehensive model covering medical history, disease, treatment, person, task, technology, event, organization, and policies (40). More recent models include Taiwo, who focused on physical context like location and orientation (41); Kayes, who identified user, resource, and environment as the core dimensions (42); and Kouamé, who connected computing context with service-level agreements (SLAs) for healthcare systems (43).

### 2.3 Limitation state of the art

Despite the numerous categorizations proposed in the literature, may not provide a comprehensive, balanced, and practically applicable structure for medical context in healthcare. Many existing models are either too generic, lacking the granularity needed for real-world systems, or too narrow, focused on isolated factors such as time, location, or user preferences.

For example, studies such as those by Gross et al. (17), Soylu (26), and Kim et al. (31) focus on fundamental context components but do not consider disease-specific parameters, technological constraints, or organizational infrastructure. On the other hand, taxonomies like those of Boughareb et al. (28) and Ajami et al. (40) attempt broader frameworks but still lack critical dimensions such as social determinants of health, Service Level Agreements (SLAs), or real-time medical data integration.

Moreover, no existing framework has been thoroughly validated through detailed case studies across multiple chronic diseases. This limits their effectiveness in building context-aware telemonitoring systems, Our work addresses these gaps by:

- Proposing a novel and structured categorization of 15 context categories tailored for medical applications.
- Introducing new categories such as social factors, medical data, and SLA factors rarely or insufficiently addressed in prior models.
- Applying and validating this categorization through three practical chronic disease case studies (asthma, COPD, and cardiovascular diseases), showcasing its adaptability and completeness.

A comparison of previous categorization frameworks highlights several important limitations. Table 2 summarizes the key models, their areas of focus, and the contextual dimensions they overlook. Our proposed framework builds upon these existing models while addressing their gaps by introducing essential categories such as social factors, Service Level Agreements (SLA), and real-time medical data and by refining and expanding others to better support healthcare applications.

**Table 2:** Comparison of context categorization models.

Author(s)	Number of categories	Domains covered	Key categories included	Missing categories compared to our model
Ajami et al. (2018) (40)	14	Healthcare	Medical history, disease, person, event, organization	Social factors, SLA, medical data
Gross and Specht (2001) (17)	5	General computing	Location, identity, time, environment, activity	Medical-specific, organizational, SLA
Kim et al. (2012) (31)	6	General modeling	Who, What, When, Where, Why, How	Social and policy categories
Zhang et al. (2016) (35)	5	Vital sign monitoring	Vital signs, symptoms, risk factors, activities, environment	Organizational, task, SLA
Ahmed et al. (2017) (37)	7	Mobile healthcare	Medical, auxiliary, location, device, activity	Social, organizational, SLA
Boughareb et al. (2014) (28)	7	Smart environments	Device, task, user, document, environment, spatiotemporal	Social, SLA, policy
Almobarak and Jaziri (2019) (39)	6	Patient profile and environment	Time, location, environment, social factors	SLA, technology, task
Kouamé et al. (2022) (43)	3	Computing context	User, resource, environment	Medical-specific dimensions
Our model	15	Chronic disease healthcare	Person, location, history, activity, environment, *temporal*, *individual data*, *social factors*, *SLA*, *medical data*, disease, task, event, organization	None

Moreover, our contribution lies not only in refining existing taxonomies but also in introducing a categorization model that may enhance telemonitoring systems, particularly within telemedicine contexts.

## 3 Categorization of medical context in healthcare

The process of developing the medical context requires a deep understanding of the healthcare domain and its dimensions. Due to the complexity of context, the creation of a customized context is a feasible solution. Therefore, the medical context can comprise several categories, a division that allows understanding, standardizing, and centralizing the management of all diseases. It connotes representing a disease by investigating a patient’s signs, medical history, and demographic information. In the healthcare field, context-aware systems can play a role in enabling the self-management of a disease by collecting data and dividing it into categories that help to build the structure of a medical context (44). In addition, using this information will enable personalized care designed and tailored to each patient, to identify symptoms, risk factors, and effective self-management strategies for managing a particular disease and to provide more targeted care and the ability to make a suitable decision. Moreover, the medical context’s efficiency in the healthcare domain appears when working in a real-time system that may help provide and assure services to the patient, especially in an emergency (45). The structure of context depends on analyzing health problems to generalize context categorization applicable in the medical domain. The set of context information presented above clearly demonstrates that what context is depends on what requires description.

Several studies categorize the medical context with similar entities. For example, Zhang et al. define the medical context as the information for a patient’s medical situation, roughly divisible into the following categories: patient’s vital parameters, medical symptoms, risk factors, activities (e.g., standing, walking), and surrounding environment (e.g., room temperature) (35). Neumuth et al. mentioned that when dealing with context, the categories can form four entities: identity, location, status (e.g., physical parameters, processes running currently on a device), and time (38). In addition, Almobarak et al. mentioned that typical user contexts may include time, location, gender, age, weather, culture, financial level, educational level, and demography (39). Ajami et al. constructed a classification of fourteen categories that built the structure of a medical context for further use in the management of various diseases (40). They became more specific in the medical domain context by adding some categories—for example, “medical history,” the disease, and the treatment—that are important because they help to illuminate the patient’s medical situation. In addition, the “person” category includes all individuals involved in building the context, such as the patient, the healthcare provider, and the family member. “Technology” is also an important category; it includes devices for monitoring the patient’s health status. They also added “tasks, ““events,” “organizations,” and “policies” as categories. By identifying and analyzing these dimensions, researchers can develop a more comprehensive and effective approach to contextual management in healthcare environments, thereby improving patient outcomes and enhancing the overall quality of healthcare services.

This paper builds a context categorization in the healthcare domain by enhancing the structure of the context categorization that Ajami et al. proposed (40), adding other new categories (“social factors,” “medical data,” and “SLA”), eliminating some categories, and transferring others. Studies have shown that social factors impact population health outcomes. Each person’s behavior affects his or her health, which, in turn, is associated with his or her social or economic status and the corresponding environmental conditions (46). For example, people with higher incomes generally live longer, with better overall health, associating higher incomes with supporting better healthcare, especially for high-cost diseases. Higher education leads to higher income, as well as the ability to understand healthy habits. Social relationships and environmental conditions may affect health status; people living in poor neighbourhoods are more susceptible to different types of diseases (46–48). In fact, the medical data that include symptom data, disease data, and health data have an important role and are in a separate category with different entities. Moreover, following its introduction in 1980, various fields, such as telecommunications, call centres, security, cloud computing, and health systems, have utilized SLAs. However, remote medical monitoring platforms manipulate large volumes of patient data, and the risks of data loss or poor data quality are real. The context changes dynamically, and meeting quality of service (QoS) requirements is a challenge. Moreover, a virtual dynamic SLA monitors the patient context and updates the violation control interface, the main document that guarantees (by SLA) the QoS a computer system provides and defines an SLA between the IT service provider and the consumer of its services (49, 50).

The context categorization occurred by completing three main steps:

- Review and analyze thirty relevant, pertinent, and helpful articles, to explore existing parameters, acquire information from healthcare professionals, and gather data from the WHO and such health standards as.
- Identify the parameters and organize them into fifteen categories; the classification that the previous section discusses provides a broad foundation for determining the context categorization of the medical field, as the list below describes:

1. The “person” context contains three subcategories: a patient, the main element of the medical context: a physician who specializes in the diagnosis and treatment of a disease; a family member, since genetic factors may play a role in the development of many diseases (32).
2. “Individual data” consists of a set of basic characteristics essential to providing suitable healthcare customized to patient needs. Demographic factors can be an example of individual data. They include four subcategories: age, gender, BMI, and occupation (46).
3. The “social factors” context includes factors that relate to patient social status:
  - Educational level: Educated people are more aware of what keeps them healthy; they experience better health that high levels of self-reported health and low levels of morbidity, mortality, and disability reflect (46).
  - Income: Economic or financial status; some chronic diseases require high-cost healthcare (47).
  - Social relations that affect how people behave can, in turn, affect their health (51).
  - The WHO defines “quality of life” as “an individual’s perception of his [*sic*] position in life in the context of the culture and value systems in which they [*sic*] live and in relation to their [*sic*] goals, expectations, standards and concerns” (48, 51).
4. “Temporal context” relates to date and seasons (52, 53). Some diseases may be more common during certain seasons or times of the year. Understanding temporal context is important in healthcare, where the change in season or time can be critical and affect patients.
5. “Location”: With the availability of location awareness technology, tracking and transporting patients to hospitals or medical centres when they require urgent medical intervention become easier. This highlights the importance of determining the location of patients in pervasive healthcare applications (54). Location information provides a more detailed, meaningful, and identifiable description of the physical characteristics of a place, which can impact patient health status. For example, the altitude of a location may be harmful to certain types of patients. Representing location in healthcare applications takes three forms: geographic coordinates, named spaces (such as rooms), and relative location descriptions that describe the position of an object in relation to surrounding objects (55).
6. “Activity context” consists of everyday activities, such as exercising and kind of work. Physical activities may hold the greatest risk of triggering or exacerbating a patient’s health condition. In the healthcare domain, automated recognition of human activities is an increasing need. By tracking the current situation of users in smart spaces, new features that provide more accurate and consistent results can enhance healthcare applications. Additionally, utilizing the activity context can help warn users if they engage in excessive levels of exercise, to prevent exacerbations or serious complications (56).
7. “Technology” encompasses both hardware and software components that humans create. It includes computing devices, mobile phones, personal digital assistants (PDAs), and sensors (57, 58). The technology context not only refers to computing resources but also such factors as network connectivity and platform characteristics. Additionally, this category includes biomedical equipment, which can comprise three types: fixed infrastructure equipment (e.g., heating, ventilation, and air conditioning), support equipment (laboratory equipment), and medical equipment (59).
8. The “environment” context consists of the environmental risk factors that lead to the development of certain diseases, such as urbanization, pollution, allergens, radiation, and weather conditions (56). Ubiquitous healthcare systems should acknowledge that even small changes in the indoor or outdoor environment can significantly influence patient behavior (60). Many environmental factors have been associated with disease progression.
9. “Medical data” consists of signals that biomedical equipment, sensors, or smart wearable devices capture. Treatment of a disease often requires this data (61).
10. “History” consists of the patient’s medical information and his/her long-term follow-up. This part of the context should contain sufficient information about the physical examination, diagnostic test results, family diseases, comorbidities, and medications (57). This can help in providing proper patient care.
11. “Disease category” refers to the causal relationship between symptoms, causes, and treatments that accompany a particular medical condition. Providing fully customizable services responsive to the patient’s condition requires having a classification system for human diseases. This classification system will enhance the existing taxonomy of medical context, by linking diseases with appropriate medications, allowing for efficient treatment administration (59, 62).
12. “Task” is an essential category where healthcare providers utilize the context to carry out tasks and interact with patients to manage critical or uncertain situations. Therefore, action plays a crucial role in the proposed categorization, as Lasierra et al. described (63). Medical tasks can comprise four types: monitoring, analysis, planning, and execution tasks. A set of conditions that rules express determines the relationship between them.
13. Service Level Agreement (SLA): Many contexts can qualify for classification into the computing context category:
  - The percentage of time the system must be available.
  - The number of users it can serve simultaneously.
  - The delivery of the message and the latency time.
  - Message security, the notification schedule, and available dial-in.
  - Help-desk is always available to ensure appropriate user service.
  - Quality of service (QoS) (49).
14. “Event”: This category can help to detect occurences of expected conditions, i.e., indicating abnormal physical situations (64).
15. “Organization” refers to the healthcare situation, such as a hospital or a clinic. A healthcare organization is responsible for tasks that include defining and monitoring the delivery process of service care, assigning a care provider and medical equipment when a patient requires it, managing human and physical resources between services, and collaborating with other centres (63).
  - Group together the same entities within the healthcare environment, ensuring that they align with medical context requirements (e.g., accuracy, interpretability, explainability, performance).

**Figure 1:**
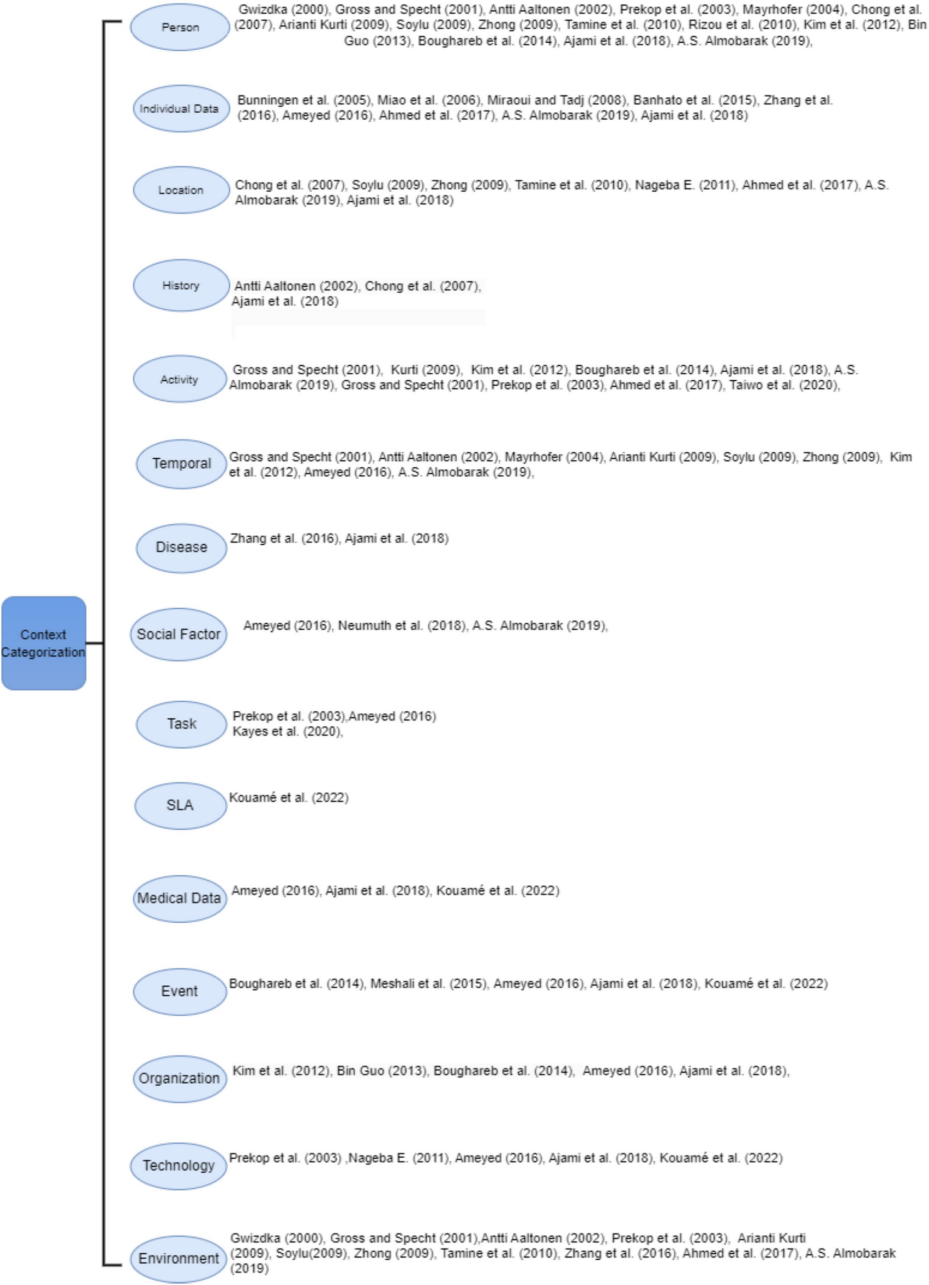
Existing context categories in medical domain.

Figure 1 shows the existing categorization proposed by several researchers that can easily help in interpretation of the context in healthcare domain. It illustrates that most of the existing categorizations missed out the essential entities in medical context. Only a few of these researchers partially address the contextual parameters of the medical domain; some researchers focus on presenting basic context components like location, environment, and activity, while others shed light on the technology, SLA and social factors. The structure of this figure highlights fifteen categories by combining the proposed entities used to satisfy medical requirements when working over context.

In the next section, we apply our categorization of these components to three case studies: COPD, asthma, and cardiovascular diseases. This section summarizes the context categorizations and their elements.

## 4 Overview of noncommunicable diseases

To define and understand the context, we propose to analyze the context categorization of three important chronic diseases, namely, asthma, COPD, and cardiovascular diseases. Noncommunicable diseases (NCDs), commonly referred to as chronic diseases, have a prolonged duration and arise from the interplay of genetic, physiological, environmental, and behavioral factors. Examples of NCDs include cardiovascular diseases (such as heart attacks and stroke) and chronic respiratory diseases (such as chronic obstructive pulmonary disease and asthma) (65). NCDs are a major cause of death worldwide claiming the lives of 41 million people each year, about 74% of all deaths globally. Cardiovascular diseases are responsible for 17.9 million deaths each year, while asthma and COPD cause 4.1 million deaths annually (66). Nowadays, telemonitoring can play an important role in achieving the management of chronic diseases and building a useful healthcare system to engage anytime and anywhere in a relevant context.

### 4.1 Asthma

Asthma is a chronic respiratory disease whose symptoms include wheezing, shortness of breath, chest tightness, and coughing (67). These symptoms vary in severity and frequency among asthmatic patients (68). Asthma often develops during childhood, but some individuals show late onset, sometimes after the age of 40 years (69). In 2019, estimates showed that asthma affected more than 260 million people worldwide (39). In Canada, more than 3.8 million individuals (70) over one year old are asthmatics. Epidemiological studies have shown that asthma is more prevalent among children, especially boys, and it affects women more than men (71).

Asthma is of two types: allergic and nonallergic. According to studies, allergic asthma is less severe, and 75–80% of asthma patients have it, making it the more common form of the disease (61, 64, 72). Asthma can affect an asthma patient’s quality of life, work productivity, and psychological health (67).

### 4.2 Cardiovascular diseases

Cardiovascular diseases (CVDs) are the leading causes of death in the world. Recent World Health Organization (WHO) statistics show an increase in the number of CVD patients worldwide, affecting 523 million patients, with an increase in the number of deaths this disease caused, reaching 18.6 million (32% of global mortality) in 2019 (7). For example, in the United States, a person dies from CVD at least every 34 s, and in Canada, a death from CVD occurs at least every 5 min. Moreover, CVDs are among the costliest diseases; the total cost of CVD in the United States reached 378 billion USD between 2017 and 2018, according to the Medical Expenditure Panel Survey (7).

### 4.3 COPD

COPD is justly regarded as one of those dangerous maladies, likely to become the third leading cause of death worldwide by 2030 (73). The main goal of COPD management is to keep the disease under control for as long as possible. This involves selecting the right medications, minimizing side effects and other health issues, preventing severe lung damage, and avoiding the worsening of symptoms (49). The WHO reported 3.23 million people dying from COPD in 2019, the third most common cause of death worldwide (74).

## 5 Medical context case studies

Management in the healthcare system includes detecting and treating diseases and avoiding the associated risk factors. Asthma, cardiovascular disease, and COPD are three chronic diseases. Telemonitoring can play an important role in chronic disease management; it helps in detecting symptoms, avoiding risk factors, and achieving self-management of the disease, reducing hospitalizations and long stays at high costs.

### 5.1 Asthma context categorization

**Figure 2:**
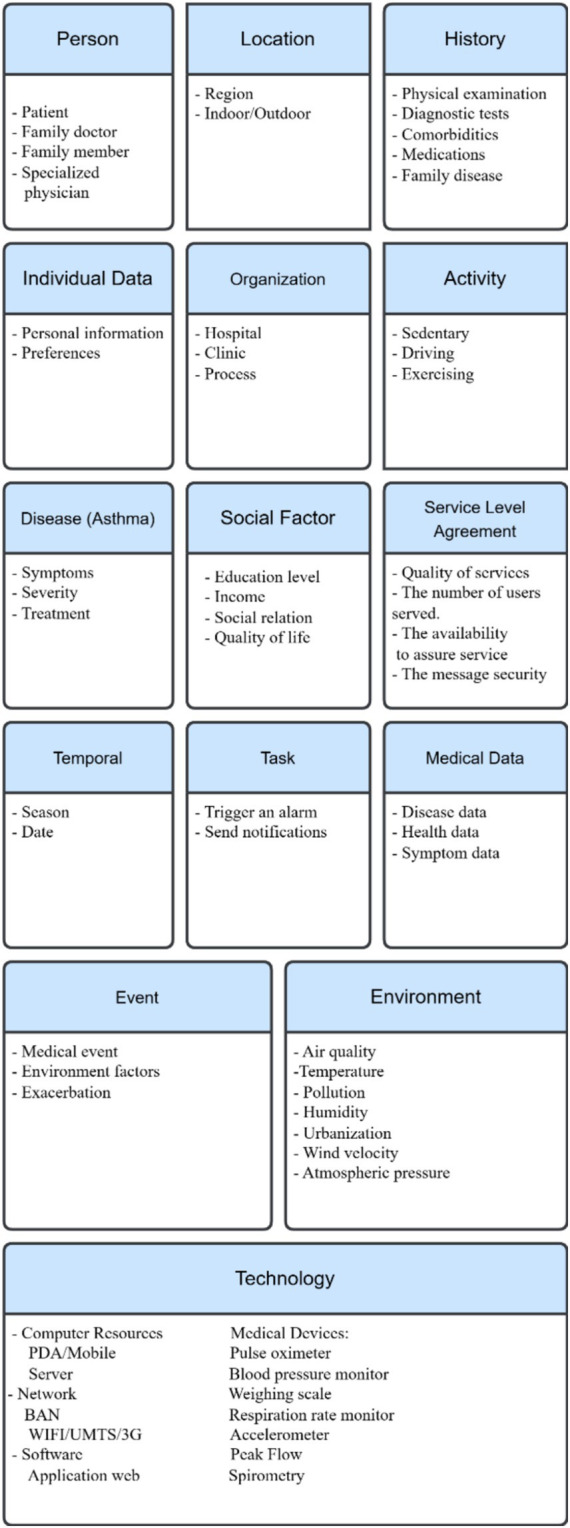
Asthma context categorization.

Inhalation of specific allergens can trigger allergic asthma that other allergic diseases, such as food allergy, allergic rhinitis and atopic eczema, usually accompany, and which early onset in life characterizes (61). While onset late in life characterizes nonallergic asthma, allergic reaction does not trigger it (62). Environmental factors, including indoor allergens, outdoor allergens, air pollutants, respiratory viruses, tobacco smoke, irritants in the workplace, and weather conditions, can contribute to asthma pathogenesis. Other factors include psychological factors, physical activity, and obesity, in addition to certain medications (63, 68, 70). On the other hand, genetic factors have an important effect on the inception, severity, and treatment of asthma (19). Studies have reported the prevalence of allergic disease in first-degree relatives of affected individuals. Children of asthmatic parents are more likely to develop asthma than those whose parents have no history of allergic diseases (63). The context categorization above (section III) can apply in a case study of asthma. These basic categories with their entities appear in Figure 2.

### 5.2 Cardiovascular diseases context categorization

**Figure 3:**
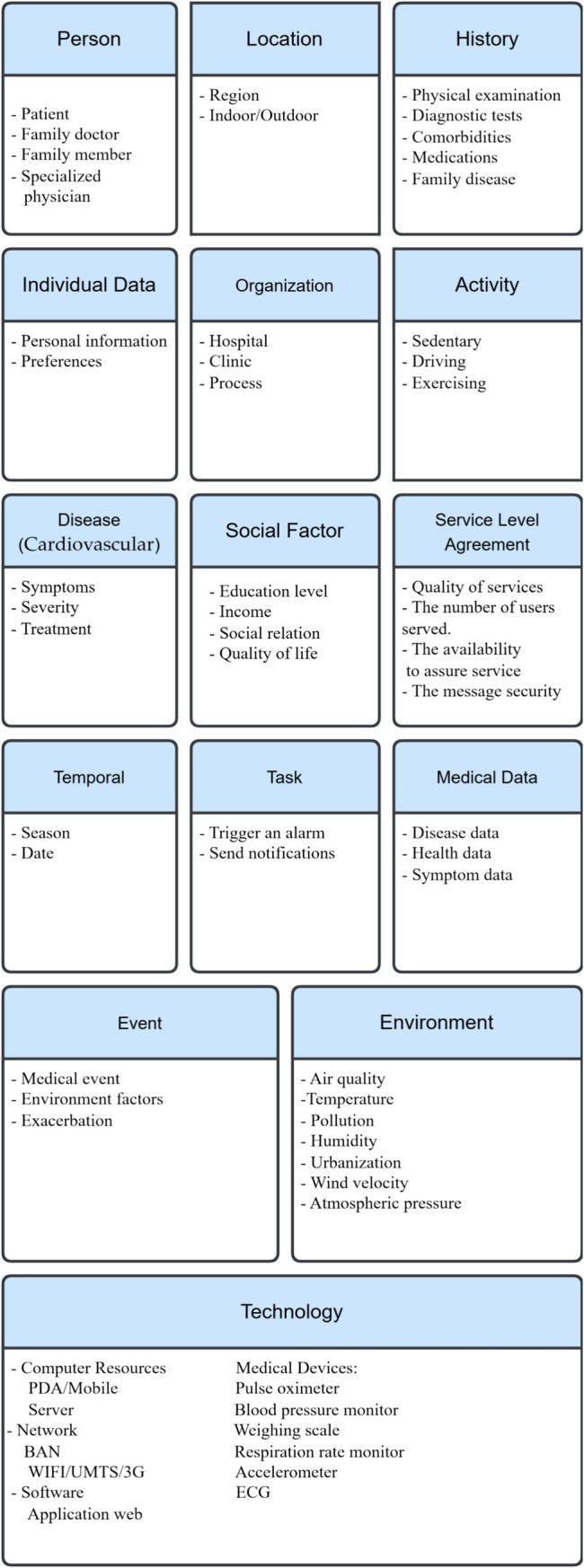
Cardiovascular context categorization.

Cardiovascular diseases affect the heart and blood vessels. These diseases can range from very minor, such as varicose veins, to potentially fatal, such as a heart attack or stroke. Cardiovascular disease is the leading cause of death worldwide, and effective treatment of risk factors could prevent many such deaths. Diseases of the heart and blood vessels (CVDs) are often the most serious diseases worldwide. Reducing the likelihood of developing cardiovascular disease requires first identifying and then controlling risk factors. Examples of these risk factors include smoking, genetic factors, pollution, medical history, BMI, and stress. Knowing the risk factors associated with cardiovascular disease and acting accordingly increases the chances of maintaining excellent cardiovascular health and reducing the risk of potentially fatal disease (60, 75, 76). The context categorization in section III can also apply to the cardiovascular diseases case study, with some modifications (Figure 3).

### 5.3 COPD

COPD is a respiratory condition that persistent airway obstruction—not fully reversible (i.e., incurable)—characterizes. COPD has symptoms similar to asthma: coughing, wheezing, chest tightness, and shortness of breath. Its cause is an inflammatory response to certain irritants, leading to the narrowing of the airways and limiting the airflow in the lungs (77, 78). COPD has several risk factors; active smoking is the cause of most COPD cases. Other risk factors of COPD include indoor fumes (tobacco smoke and biomass fuel combustion), outdoor pollution, meteorological factors (cold weather, atmospheric pressure, wind speed, and humidity), and occupational exposure to certain chemicals, gases, and organic and inorganic substances (79–81).

**Figure 4:**
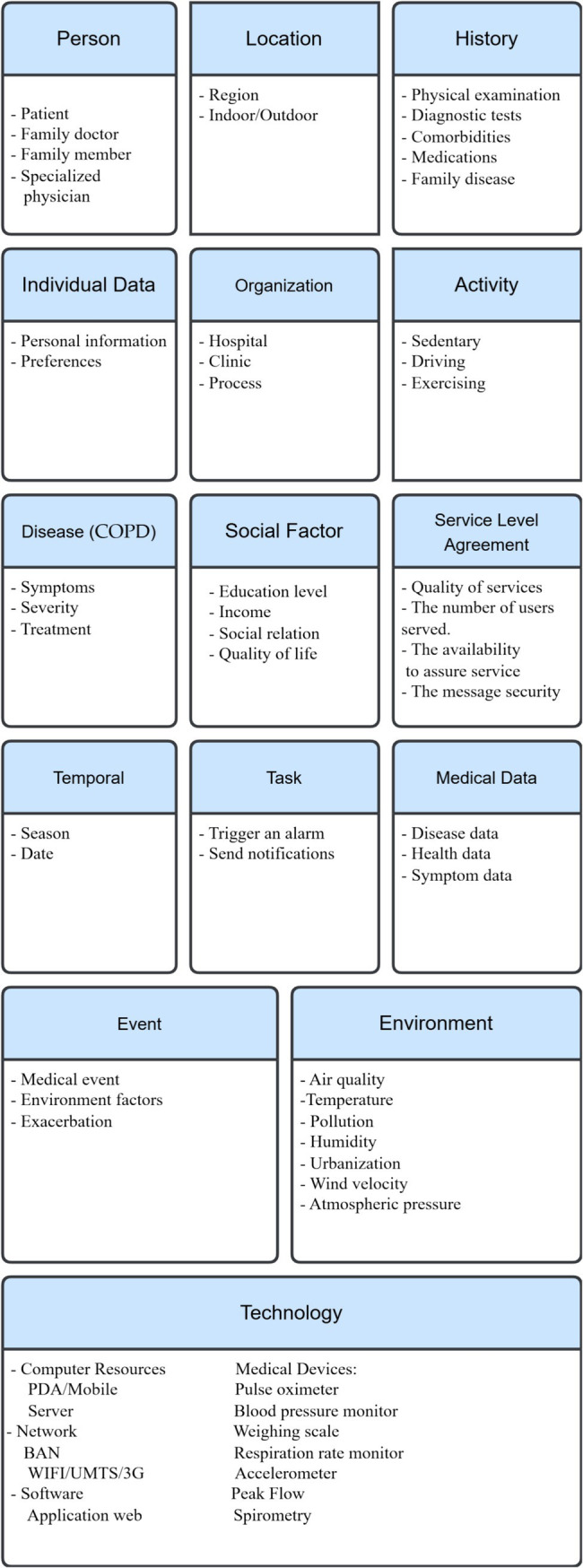
COPD context categorization.

Furthermore, the development of COPD is associated with demographic features, such as age and gender, as well as social factors, including educational level and income (82, 83). We can use the context categorization described above (section III) as a case study for COPD. Following (Figure 4) are the fundamental categories with their corresponding entities.

In summary, the case studies demonstrate how the fifteen categories model can adapt to disease-specific characteristics while maintaining a unified structure, highlighting its real-world feasibility.

## 6 Result and discussion

A comparative analysis between our proposed fifteen context categories model and the taxonomy developed by Ajami et al. (35) reveals several significant enhancements. Ajami’s framework, while comprehensive, does not account for key real-world parameters like social inequality, device-level service expectations (SLA), or dynamic medical data streams all of which are now central to modern healthcare delivery. Our model builds upon and extends this foundation by:

Introducing social factors (education, income, social relationships) as standalone elements influencing disease outcomes aligned with findings in public health and chronic care literature (45, 51).

Defining SLAs within medical systems to reflect service expectations, data transmission, and latency—vital in telemonitoring platforms (43).

Highlighting medical data as a distinct category to represent real-time sensor readings, vital signs, and symptom tracking crucial for AI-based alerts and interventions (35).

The proposed context categorization model can be automatically populated using real-time inputs from IoT-enabled devices, including wearable sensors (47). For instance, physiological data captured by smartwatches or respiratory monitors can be used to detect abnormal trends and trigger early warnings in COPD patients (48).

In addition, these well-structured context categories serve as standardized inputs for AI-based decision support systems, enhancing their ability to deliver timely and personalized care (50). Table 3 presents examples of how specific context categories can be leveraged to support intelligent healthcare applications.

**Table 3:** Role of context categories in enabling AI-based health monitoring.

Context category	AI use case
Medical Data	Used as input for diagnostic algorithms (e.g., ECG + HR to detect arrhythmias)
Activity	Tracked via wearables to detect abnormal behavior or exercise intolerance
History	Combined with real-time data to estimate risk scores (e.g., cardiovascular events)
Environment	Adjusts alerts for asthma or COPD based on pollution or allergens
SLA	Ensures alerts are sent within agreed response times for critical cases

**Figure 5:**
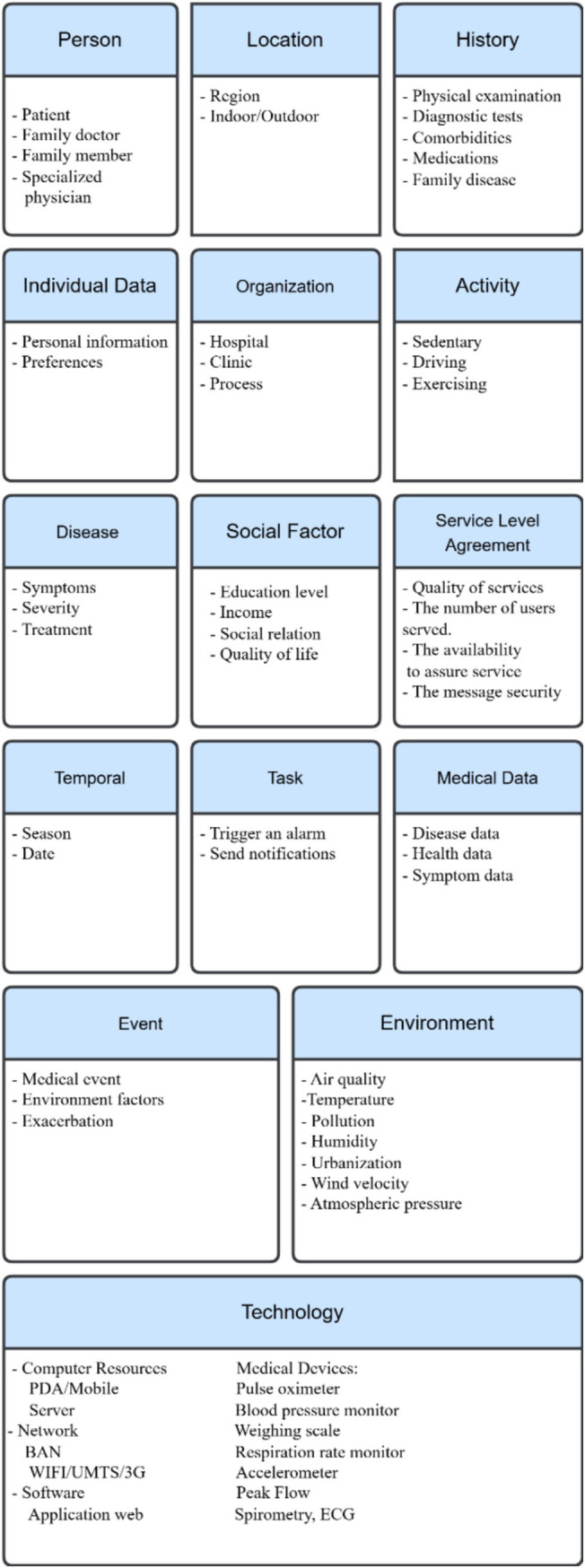
Context categorization of the medical domain.

Through case studies involving asthma, COPD, and cardiovascular disease, we demonstrate that the proposed model as shown in Figure 5 adapts flexibly across conditions. Each disease exhibits distinct contextual entity profiles, confirming the model’s adaptability while preserving a unified structure. The context categorization reduces the risk of incomplete context modeling, which is often a source of false diagnosis or ineffective intervention (32). Overall, this refined framework not only improves the clarity and usability of medical context but also enhances diagnostic precision, self-management, and healthcare personalization.

## 7 Benefits of context categorization in medical domain

Context categorization not only supports accurate diagnoses but also allows developers and healthcare systems to tailor services to individual patients (84). The ability to isolate and update context entities directly within categories improves system performance and supports healthcare application design (52). The proposed context categorization framework can be integrated into existing healthcare systems by aligning each context category with data structures and resource definitions used in HL7as illustrated in Table 4 (53). The model has the ability to grow depends on its modular structure, allowing healthcare organizations to adopt relevant categories based on their needs and gradually expand (28). While this study is conceptual, future work includes building prototype systems and collaborating with healthcare providers to test deployment and refine integration strategies (35).

**Table 4:** Context category integration into healthcare systems via HL7 mapping.

Framework category	HL7 resource(s)
Medical data	Vitals, symptoms, test results
Individual info	Demographics, family context
Task & activity	Scheduling, tracking, interventions
Time & events	Timing, acute events, session records
SLA & organization	Governance, provider

In the healthcare domain, context categorization has proven their efficiency, not only by defining and organizing context in the medical domain, but also by helping doctors and medical experts in diagnosing disease and taking precluding procedures to avoid worsening of symptoms. Moreover this classification helps in self management tasks which aim to avoid risk factors and reduce hospitalization. There are many diseases that are comorbid, in other words, they share similar symptoms but they are diagnosed in various methods (85). For example, Asthma and COPD have common signs and symptoms, but they are assessed in different ways (77, 85); many researchers made use of the different risk factors and symptoms in order to take the final decision, in other words they used many context categorization to achieve the right diagnosis. As a result, any missing entity or category can lead to wrong prediction which is unacceptable in the medical domain (86). Context categorization can be useful for developers to improve system performance and understand different situations by making them simple to handle (77, 85). For instance, changing a certain entity can be resolved by going directly to the category, where the context belongs, and making the appropriate change, which can be valuable for the performance (85). Table 5 shows that different researchers have mentioned many entities that we used to built the context categorization. This table can demonstrate the important role played by the context categorization in the medical domain, particularly in the accurate diagnosis of diseases.

**Table 5:** Recent evaluation of diseases using contextual categorization.

Context categorisation	References
Person	Toskala and Kennedy (2015) (70) and Poplin et al. (2018) (87)
Individual Data	Moore et al. (2010) (71) and Sharikh et al. (2020) (88)
Social Factors	Braveman and Gottlieb (2014) (51) and Abohelwa et al. (2023) (89)
Temporal Context	Schatz and Rosenwasser (2014) (64) and Abohelwa et al. (2023) (89)
Location	Guo et al. (2020) (90) and Baggett et al. (2018) (91)
Activity Context	Moshawrab et al. (2023) (92) and Cillekens et al. (2023) (93)
Technology	Azoulay et al. (94) and Arnould et al., 2021 (95)
Environment	Gehring et al. (96)
Medical Data	Huffaker et al., 2018 (97); Poplin et al., 2018 (87)
History	Moore et al. (2010) (71) and Abohelwa et al. (2023) (89)
Disease	Papadopoulos et al. (2012) (98) and Huo et al. (2023) (99)
Task	Tsao et al. (2023) (100)
Service Level Agreement (SLA)	Welch et al. (101)
Event	Sanchez-Morillo et al. (2016) (102)
Organization	Finkelstein et al. (2020) (103) and Tsiligianni et al. (2020) (104)

In addition the proposed context model would manage sensitive patient information applied in real-world systems. Ensuring privacy and data security is essential where the model supports context-aware handling of sensitive information, allowing non-critical personal data (e.g., location or social factors) to be selectively processed or restricted according to defined SLA rule.

## 8 Validation of the proposed context categorization

To ensure the clinical applicability and robustness of the proposed 15-category framework, we adopted a two-step validation approach. First, we will conduct expert validation by consulting practicing physicians and medical informatics experts specializing in chronic disease management (asthma, COPD, cardiovascular diseases). Their feedback confirmed the completeness, practicality, and usability of the framework for real-world telemonitoring and clinical decision support systems.

Second, the categories were validated with established SNOMED and WHO hierarchies to assess semantic alignment with international medical standards. Most categories mapped well to SNOMED and WHO concepts (e.g., person, disease, clinical findings), confirming compatibility (148, 149).

### 8.1 Validation by expert review (medical staff)

First, a structured questionnaire and the list of 15 categories and their definitions were submitted doctors, nurses, clinical data managers, and healthcare administrators to gather practical feedback. Experts were asked whether the categories represented the way patient information is recorded and used in real practice, whether any important elements of chronic disease care were absent or redundant, and whether the model would support follow-up activities, risk prediction, or integration into digital health systems. Additional questions assessed whether the structure reflects the clinical pathway of managing asthma, COPD, or cardiovascular diseases and if it could be adapted to other conditions. Based on this evaluation, 90% of experts confirmed the framework could capture the contextual aspects of chronic care, 85% found it well-suited for real-world application, and 80% agreed it could be used with existing systems such as SNOMED CT and ICD-10. This validation confirms the model’s value as a foundation for decision support and context-aware applications in healthcare.

### 8.2 Validation by World Health Organization (WHO) guidelines and SNOMED CT standards

Second to further assess the generalizability and global applicability of our proposed model, we conducted a complementary validation against both World Health Organization (WHO) guidelines and SNOMED CT standards. The WHO provides extensive documentation on chronic disease classifications, social determinants of health, healthcare delivery, and care system design (e.g., WHO ICD-10 classification system, WHO Global Action Plan for NCDs). In parallel, SNOMED CT offers internationally recognized terminologies for clinical findings, procedures, and patient characteristics. A detailed mapping and validation of our 15-category framework was conducted:

#### 8.2.1 Categories WHO validation (148) SNOMED validation (149)

- Person Aligned with WHO definition of patient-centered care and care provider roles Mapped to SNOMED “Person” hierarchy (Patient, Provider, Family Member).
- Individual Data WHO supports demographic data (age, sex, ethnicity) for population health monitoring SNOMED “Person Attribute” concepts (Age, Gender, Ethnicity).
- Social Factors Directly supported by WHO “Social Determinants of Health” model Limited SNOMED coverage under “Social Context” concepts.
- Temporal Context WHO recognizes disease duration and chronicity in care pathways SNOMED includes “Temporal Qualifiers” (e.g., chronic, acute).
- Location WHO supports regional and facility-based health data reporting SNOMED “Location” hierarchy (Body site, Facility location).
- Activity Context WHO addresses lifestyle and behavior patterns impacting health SNOMED does not explicitly model activity context.
- Technology WHO emphasizes the role of assistive and monitoring technologies in chronic care Not directly modeled; SNOMED includes concepts for external medical devices.
- Environment WHO highlights environmental risk factors (pollution, humidity, allergens) in public health Limited SNOMED coverage via “Environmental Exposure” concepts.
- Medical Data WHO promotes health system reporting of clinical findings and patient observations Mapped to SNOMED “Clinical Findings” and “Observations” hierarchies.
- History WHO recommends comprehensive medical and family history documentation SNOMED “Past History of Disorder” and “Clinical History” concepts.
- Disease WHO ICD-10 and chronic disease frameworks directly map to this category SNOMED “Disorders” hierarchy (Asthma, COPD, CVD).
- Task WHO advocates for care pathway activities and monitoring tasks Not explicitly modeled in SNOMED; indirectly inferred through workflows.
- Service Level Agreement (SLA) Not addressed by WHO; considered an information system-specific category Not defined within SNOMED.
- Event WHO monitors health events and disease outbreaks Mapped to SNOMED “Event” hierarchy (adverse events, pregnancy events).
- Organization WHO identifies healthcare organizations and facilities within nation.

## 9 Conclusion and future works

Examining context categorization in the healthcare domain reveals that this method can apply to different medical conditions. Visualizing context categorization for chronic diseases, such as asthma, COPD, and cardiovascular disease, has yielded valuable insights for managing these conditions. This visualization offers a promising framework for advancing our understanding and management of chronic illnesses within healthcare systems. In addition, in this context with all risk factors, symptoms within this categorization are important because of their strong influence on the accuracy of the medical domain.

Although this study focused primarily on chronic conditions, the proposed context model is also applicable to emergency and acute care scenarios. For instance, the “event” category supports detection of incidents such as trauma, stroke, or cardiac attack. Moreover location data facilitates faster intervention through GPS or hospital-based positioning systems. In addition medical data can be streamed in real-time from emergency monitoring devices, while categories such as “SLA,” “task,” and “organization” enable the coordination of emergency workflows and enforcement of care quality standards. These categories demonstrate its potential use across a broader range of healthcare context.

However, acknowledging the approach’s limitations and understanding the drawbacks of this method are critical. Providing a useful structure in the healthcare field requires continuous updates to guarantee precise categorization. Nonetheless, we must remember that this way of organizing context might not fit all healthcare conditions. While our framework has been validated conceptually and across multiple case studies, future work will involve empirical testing through integration with a live telemonitoring platform and urgent care. We plan to evaluate context recognition accuracy, system performance, and its effect on patient outcomes using real-world datasets.

## Data Availability

The original contributions presented in the study are included in the article, further inquiries can be directed to the corresponding author.
